# Socioeconomic status and hospital utilization among younger adult pneumonia admissions at a Canadian hospital

**DOI:** 10.1186/1472-6963-6-152

**Published:** 2006-11-25

**Authors:** Margaret J McGregor, Robert J Reid, Michael Schulzer, J Mark Fitzgerald, Adrian R Levy, Michelle B Cox

**Affiliations:** 1 Centre for Clinical Epidemiology and Evaluation, Family Practice Research Office, 828 West 10^th ^Avenue, Vancouver, BC, Canada; 2 Department of Family Practice, Department of Medicine, University of British Columbia, 5950 University Boulevard, Vancouver, BC, Canada; 3 Department of Health Care and Epidemiology, Faculty of Medicine, University of British Columbia, 5804 Fairview Avenue, Vancouver, BC, Canada; 4 Group Health Cooperative, 1730 Minor Avenue, Suite 1600, Seattle, WA, USA; 5 Centre for Health Evaluation & Outcome Sciences (CHÉOS), St. Paul's Hospital, 620-1081 Burrard Street, Vancouver, BC, Canada

## Abstract

**Background:**

Although the general association between socioeconomic status (SES) and hospitalization has been well established, few studies have considered the relationship between SES and hospital length of stay (LOS), and/or hospital re-admission. The primary objective of this study therefore, was to examine the relationship of SES to LOS and early re-admission among adult patients hospitalized with community-acquired pneumonia in a setting with universal health insurance.

**Methods:**

Four hundred and thirty-four (434) individuals were included in this retrospective, longitudinal cohort analysis of adult patients less than 65 years old admitted to a large teaching hospital in Vancouver, British Columbia. Hospital chart review data were linked to population-based health plan administrative data. Chart review was used to gather data on demographics, illness severity, co-morbidity, functional status and other measures of case mix. Two different types of administrative data were used to determine hospital LOS and the occurrence of all-cause re-admission to any hospital within 30 days of discharge. SES was measured by individual-level financial hardship (receipt of income assistance or provincial disability pension) and neighbourhood-level income quintiles.

**Results:**

Those with individual-level financial hardship had an estimated 15% (95% CI -0.4%, +32%, p = 0.057) longer adjusted LOS and greater risk of early re-admission (adjusted OR 2.65, 95% CI 1.38, 5.09). Neighbourhood-level income quintiles, showed no association with LOS or early re-admission.

**Conclusion:**

Among hospitalized pneumonia patients less than 65 years, financial hardship derived from individual-level data, was associated with an over two-fold greater risk of early re-admission and a marginally significant longer hospital LOS. However, the same association was not apparent when an ecological measure of SES derived from neighbourhood income quintiles was examined. The ecological SES variable, while useful in many circumstances, may lack the sensitivity to detect the full range of SES effects in clinical studies.

## Background

Modern epidemiology studies have established a clear association between socioeconomic status (SES) and health status even after standardization for all known confounders. Low SES has been shown to be an independent predictor of higher mortality rates [1,2], higher disease prevalence [3,4], higher hospitalization rates [5-7], and poorer treatment response and prognosis [8,9] for a wide range of illnesses across many countries with differing health care systems [10].

Far fewer studies have examined the relation between hospital length of stay (LOS), as a measure of health services utilization, and SES. Some US studies have found LOS to be inversely related to SES [11]; others have found no effect [12]. In one study that examined the relationship between race and hospital LOS among the elderly, African-Americans were found to have a significantly shorter LOS after adjusting for age and health status [13]. In Canada, Brownell and Roos found a small inverse association between neighbourhood-level income quintiles, an ecological indicator of SES, and LOS for patients admitted to eight Manitoba hospitals for 14 common illnesses between 1989 and 1992 [14]. In contrast, Glazier and colleagues found that once admitted to hospital, there was no relationship between neighbourhood-level income quintiles and LOS [15]. We are unaware of any Canadian studies that have examined individual measures of SES in relation to hospital LOS.

These contrasting findings may be explained by a number of factors. First, some studies are limited by their use of ecological measures of SES, resulting in misclassification and potential bias toward the null, especially for smaller effects. With a greater mix of individuals at differing levels of SES in a particular neighbourhood, this misclassification will be greater. Second, inconsistent results may be due to differences in adjustment for important potential confounders beyond clinical case mix (for example, level of function and living situation). Finally, the impact of SES on health services utilization is likely to be influenced by access to and co-payments for hospitalization, and studies from countries with differences in health care access and insurance arrangements may produce different results. Hofer et al. found that SES effects on hospitalization were substantially diminished when they controlled for insurance and health status [16].

In order to understand the impact of SES on LOS, it is also important to examine early re-admission. This measure is less frequently examined in relation to SES, and yet is crucial in understanding whether hospital stays are meeting the needs of different socioeconomic groups equitably. The purpose of this study was to examine the relationship of SES to hospital LOS and early re-admission for adults less than 65 years old admitted with community-acquired pneumonia in a health care system with universal insurance for hospital and physician care. We examined pneumonia because it is one of the most common reasons for medical admission to hospital throughout the Western world [17]. It was hoped that by performing individual-level adjustment using broader clinical data we could begin to clarify the complexities of the association between SES and hospital utilization.

## Methods

### Study setting and population

Canada has a publicly funded health care system providing residents with universal insurance for medically necessary health care. Patients may present to any acute care hospital and receive first dollar coverage for the care received. Although some private facilities have opened for certain types of surgical services, there are no private acute care hospitals to treat medical conditions. The Vancouver General Hospital (VGH) is a large teaching hospital situated in central Vancouver, British Columbia, which serves patients from a large geographic area including both poor and wealthy neighbourhoods. This study was a retrospective longitudinal cohort analysis of adult patients randomly selected from the total 3,934 admissions to Vancouver General Hospital who had a most responsible diagnosis of community-acquired pneumonia (ICD-9-CM codes 481.XX – 483.XX, 485.XX, 486.XX) between January 1, 1990 and March 31, 2001. Repeat admissions on the same individual were eligible for inclusion at the time of the index admission and excluded thereafter.

Included for computer-generated random selection was any individual admitted for pneumonia, living in the community of Vancouver or Richmond, with a valid British Columbia Medical Services Plan number who was discharged alive (n = 3,934). We excluded those admitted from or discharged to another acute care hospital in the province (n = 146); outliers with an LOS greater than 3 times the inter-quartile range (n = 13); or those who left the hospital against medical advice (n = 57). Ethics approval was obtained from the Ethics Board of the University of British Columbia and the Vancouver Hospital and Health Sciences Centre Research Advisory Committee. In this study we present results on all reviewed charts from patients less than 65 years old.

### Data sources/data collection

Data were obtained from four sources that were linked, at the individual level, using patient-specific identifiers.

1) A computerized hospital discharge abstract database, used to define the cohort from which admissions were randomly sampled, provided information on age, sex, 6-digit postal code of residence, co-morbid diagnoses and hospital LOS.

2) A Statistics Canada postal code conversion program [18,19] linked individuals' postal codes to enumeration area census data on mean household income.

3) Structured chart review of sampled admissions by two registered nurses with extensive chart review experience, gathered individual-level data on demographics, illness severity, functional status and other measures of case mix.

4) The BC Linked Health Database, an individual-level, population-based research resource of linkable health care utilization and other data, provided data on all re-admissions for any cause to any BC hospital within 30 days of discharge from the sampled hospitalization. This database is developed and maintained by the Centre for Health Services and Policy Research at the University of British Columbia, in collaboration with the British Columbia Ministry of Health [20].

### Data measures

#### Length of stay (LOS), re-admission and socioeconomic status (SES)

Hospital LOS was measured in days from the date of admission to the date of discharge. Our second outcome of interest was re-admission to any acute care hospital in the province of British Columbia for any cause within 30 days of discharge from the index admission.

SES was measured in two ways:

1) Individual-level financial hardship measured by receipt of income assistance or provincial disability pension

To qualify for income assistance provided by the Government of British Columbia, a person's time-limited employment insurance must have expired and they must have depleted their savings. In 1996 (the approximate midpoint of the study period), the annual income of a single BC resident on income assistance was C$7,081 [21]. To qualify for a provincial disability pension, an individual must prove a longstanding physical or mental disability and be ineligible for a federal disability pension. The annual income for a single person in BC living on a provincial disability pension in 1996 was C$10,784 (Table 1) [21]. An individual is not eligible to receive income assistance if they are receiving a disability pension, and eligibility for either of these benefits applies only to those less than 65 years old.

This variable was derived from chart review data and coded as "yes" if there was a chart notation that the patient collected income assistance or received a disability pension. This information was recorded by clerical staff on the admission face sheet at the time of admission, under the heading of "employer" and supplemented with social worker and clinical service providers' notes.

2) Neighbourhood-level income quintiles

Patient residential postal codes were aggregated into census enumeration areas based on the 1996 definitions using software provided by Statistics Canada [18,19]. Incomes (defined as income per single person equivalent) were based on census data on average household income and the distribution of households by size in each enumeration area. Within the enumeration areas represented by the study population, the lowest income ranged from C$10,950 to C$29,001 (Table 1). While not an individual-level measure of income, the use of this technique has been found to correlate well with individual SES in large population-based analyses and has been used extensively in health care research as a surrogate measure for SES [16].

#### Other variables examined

We examined age, sex, smoking status and substance dependency as potential confounders. An individual was considered to be a smoker if there was any history of smoking documented in the year prior to admission. Substance abuse was coded as "yes" if there was documentation of alcohol, cocaine, heroin, prescription or illicit substance dependency at the time of admission.

We also measured pneumonia severity, using chart review data to construct a "Pneumonia Severity Index" (PSI) [22]. This index is calculated from individual demographic (age and sex), disease co-morbidity (neoplastic disease, hepatic disease, renal failure, congestive heart failure and cerebrovascular disease), and a mix of physiologic and laboratory measures. The index has been used clinically to determine when pneumonia patients should be hospitalized [22] and by researchers to explain variations in mortality and hospital LOS for pneumonia patients [23].

We used an imputation process to derive missing values in the PSI data. Ten percent of these data were missing and occurred at random with no relation to age. Cases with a single missing variable were filled in first. Using logistic regression on the data subset, the missing variable was regressed on all other variables in the PSI list. The derived regression was then applied to each case for which this variable was singly missing, and the predicted probability of the presence of the variable was estimated. Whenever this probability exceeded 0.5, the variable was entered as present for the case. For cases with multiple missing variables, a similar logistic technique was applied, replacing one variable at a time while omitting the other missing variables from the model, and building up the complete dataset in a stepwise manner.

The variable "living alone" was coded "yes" if a patient was classified in the chart to be living alone at the time of admission. Functional impairment or "any documented problems with activities of daily living (ADLs)" was coded as "yes" if there was documentation in the nursing notes of difficulty with feeding, mobility, bathing, dressing and/or toileting on admission. We note that one of our SES measures looked at "receipt of disability" and therefore had the potential for correlation with functional impairment. However, disability pensions are commonly provided for psychological disabilities rather than the functional impairments coded here.

We also used a modified Deyo adaptation of Charlson co-morbidities for the ICD-9-CM codes listed as contributing to hospital stay [24,25]. Secondary diagnoses extracted from the hospital discharge database were used to construct this index. We excluded co-morbidities from the Charlson index that were already captured by the PSI variable discussed above, including neoplastic disease, renal failure, hepatic disease, congestive heart failure and cerebrovascular disease.

#### Data analysis

After examining the data for outliers and possible data entry errors, we generated descriptive statistics and calculated crude mean and median lengths of stay for each study population characteristic. Appropriate parametric and non-parametric tests of comparison were used for univariate testing of LOS by the various factors. The distribution of each SES measure across other independent drivers of LOS was examined with bivariate linear regression models.

#### SES and LOS

We examined the association between individual-level (receipt of income assistance or disability pension) and neighbourhood-level (income quintiles) SES measures and LOS using two separate multiple linear regression models. Due to the skewed distribution of our outcome variable (LOS), regression analyses were carried out after logarithmic transformation of this variable. Forward and backward stepwise linear regression was run entering all measured co-variates. Variables that were not significant at p < 0.05 in the model were dropped. We exponentiated the parameters of the model to generate the estimated adjusted multiplicative effects and 95% confidence intervals for each SES variable on LOS.

#### SES and re-admission

Logistic regression models were used to examine the adjusted effect of each of our two SES measures on re-admission within 30 days. Variables significant at p < 0.10 in univariate regression analysis were initially included in the multiple regression model and then omitted from the full model if they were not significant at p < 0.05.

Finally, all models were tested for co-linearity and two-factor interaction effects. Co-linearity was tested by analyzing the correlation matrix, dropping highly correlated variables (correlation coefficient ≥ 0.8), re-analyzing with different combinations and multiple models until stable results were obtained.

## Results

### Characteristics of the study population

A total of 148 patients (34% of the population under study) had individual-level financial hardship, with 84 (19%) patients on income assistance and 64 (15%) collecting a provincial disability pension (Table 1). Forty-five percent of individuals with available data (183/410), had postal codes corresponding to the lowest neighbourhood-level income quintile and the median income quintile was the second income quintile, representing an estimated range of C$29,037 – C$33,944 (Table 1). Table 2 describes the proportion of those identified with financial hardship by neighbourhood-level income quintiles.

A higher frequency of male admissions (63% vs. 53%, p = 0.05), living alone (41% vs. 16%, p < 0.001) and identified problems in activities of daily living (7% vs. 3%, p < 0.05) was seen among those with individual-level financial hardship (Table 3). This group also had a significantly higher mean PSI score on admission (75.5 vs. 68.1, p < 0.05). In contrast, there were no significant differences in the distribution of the above variables across the lowest income quintile grouping (quintile 1) compared to quintiles 2 to 5 (Table 3).

### SES and length of stay (LOS)

Those with individual-level financial hardship had a longer median length of stay of 6 versus 4 days compared to those without – a difference that was significant in univariate testing (p < 0.01) (Table 4). After adjustment for PSI, number of co-morbidities, any problem with activities of daily living, living alone and year of admission, financial hardship was associated with an estimated 15% (95% CI -0.4, +32, p = 0.057) longer LOS (Table 5). Sex, smoking and substance abuse were not significant in the multiple regression analysis and therefore not included in the final model.

There was no significant association of neighbourhood-level income quintiles with LOS in univariate (Table 4) or multiple regression analysis (Table 5).

### SES and hospital re-admission within 30 days

Three hundred and fifty one records (81%) were successfully linked to secondary hospital discharge data. Linkage of reviewed cases admitted in the last three years of the study time period was hindered due to technical difficulties with the linkage process for those years. Among those that were linked, there were 43 (12%) all-cause hospital re-admissions within 30 days of discharge from the index admission. Twelve re-admissions (3%) occurred within the first 10 days and 22 (6%) occurred within the first 20 days. Apart from year of admission, there were no differences between linked and unlinked cases with the exception of a higher proportion of individuals in the first (lowest) income quintile among the unlinked (16/83, 53%, n = 83) compared to linked cases (139/351, 40%, n = 351).

Among those re-admitted, just over one half (53%, n = 23) had individual-level financial hardship compared to 30% (n = 91) of those not re-admitted. In the multiple regression analysis, financial hardship was associated with re-admission (adjusted OR 2.65, 95% CI 1.38, 5.09) (Table 6). Male sex was also associated with early readmission (adjusted OR 2.05, 95% CI 1.01, 4.18) in the multiple regression model.

There was no significant association of neighbourhood-level income quintiles with re-admission in either univariate or multiple regression analysis (Table 6). When we re-ran the models using income deciles vs. quintiles, results were the same. When we re-ran the models excluding cases with imputed data, there was also very little difference in the magnitude of estimated effect for either outcome. There were no significant interaction effects for any of the models.

## Discussion

Our study found that approximately one in three admissions were on social assistance or collecting disability pension. This is substantially higher than the reported average of 7.77% of the BC population collecting social assistance or disability pension between 1995 and 2000 [26]. We also found a disproportionately high number of pneumonia admissions had income quintiles in the bottom 20% of incomes in Vancouver. Both findings are consistent with the literature in a number of Western countries, that hospitalized pneumonia [27] and general medical admissions [5-7] tend to be poorer than the general population.

### Individual-level financial hardship and hospital utilization

Our study found a significant difference in median, unadjusted LOS among pneumonia patients less than 65 years old with individual-level financial hardship (6 vs. 4 days, p < 0.01). This association had marginal significance after adjustment for case mix, functional impairment, and living alone, and ultimately only a small amount of the variation in outcome was explained by financial hardship (R squared change from 23.9% to 24.5% after inclusion in the model). This suggests that once admitted to hospital with pneumonia, there is a very minor effect of financial hardship on hospital stay per se. Rather, the impact of SES is confounded by a range of other characteristics (illness severity, disease co-morbidity, functional status and living alone) that both lengthen LOS and are disproportionately present among those with financial hardship. This finding is quite different from those of Stelianides and colleagues who prospectively examined 107 patients hospitalized for pneumonia in France [27]. These authors found an adjusted 5.7 days longer LOS associated with low SES. However the mean LOS of all patients reported in this study was substantially higher (15 days) than in our study and there was no adjustment for factors beyond clinical case mix.

Our finding that those with individual-level financial hardship had a greater than 2.5-fold adjusted odds of re-admission within 30 days of discharge suggests a number of scenarios. Patients may experience destabilization of their illness following discharge due to challenging social circumstances such as poor housing and inadequate nutrition. Also, these individuals may be less likely to adhere to post-discharge treatment plans because of a decreased ability to access needed ambulatory care. Alternatively, patients with financial hardship may be more vulnerable to experiencing a new illness that is not directly related to their initial hospitalization with pneumonia.

From a health policy perspective, and regardless of the mechanism, the study results suggest that there may be insufficient provision of post-discharge services after a hospitalization for pneumonia. One might argue that there is a false economy to discharging patients who are medically stable but "socially precarious" and that failure to address the latter context results in early post-discharge medical destabilization with costly re-admission. The piloting of programs to provide "sub-acute" medical and social support, or systems that follow low income patients in the community after discharge to address potential complications, may be useful in decreasing rates of re-admission among this population. Prospective research, to better understand the causes for re-admission among those with financial hardship, and to pilot re-admission prevention programs among this population, would also be an important line of further research.

### Ecological SES (income quintiles) and hospital utilization

In contrast to the link between individual-level financial hardship and hospital LOS and re-admission, we found that the neighbourhood-level income quintile variable was remarkably inert. We suggest that this may be due to a lack of precision of this ecological variable in identifying individuals experiencing a more extreme degree of financial hardship. Seventy-eight of the one hundred and twenty-nine (60%) individuals with financial hardship also had postal codes corresponding to the lowest income quintile, and the association was significant (Pearson Chi-square = 19.09, p < 0.001). On the other hand, a substantial number of those with financial hardship (n = 51, 40%) also had postal codes that fell into neighbourhood-level income quintiles outside of the poorest quintile.

Other possible explanations for the inconsistency in results between individual and ecological measures of SES include the following. Firstly, the low income range represented by postal codes in the bottom quintile was substantially higher than the income represented by those with individual-level financial hardship (C$10,950 – 29,001 vs. C$7,081). Secondly, the group with individual-level financial hardship had a disproportionate number of missing postal codes, compared to the rest of the population under 65 years (13% vs. 6%). This may reflect a greater proportion of individuals with financial hardship and unstable housing for which a residential postal code could not be assigned. Finally, while postal code of residence may be good at identifying neighbourhood characteristics such as access to transit and green space, one would expect pneumonia to be more related to individual-level characteristics like housing conditions, nutrition and poverty.

These differences of precision and type of measure may be less important for studies with large sample sizes. Roos et al. examined administrative data for the urban population of Manitoba (N = 794,555) and demonstrated a significant inverse socioeconomic gradient for pneumonia hospitalization rates using the same postal code derived neighbourhood-level income measure used in our study [28].

This finding has important implications for health services researchers who are often restricted, by data availability, to the use of ecological measures when adjusting for SES. While the imprecision of neighbourhood-level SES measures resulting in random misclassification of observations is unlikely to attenuate SES effects in large population studies [16,29], our research suggests that they should be used with caution in smaller clinical studies. Beyond the problem of misclassification, it should be noted that ecological measures presuppose the presence of a fixed address – something that is often missing among the most marginalized patients, whose social circumstances are also most likely to affect the health outcomes examined. Exclusion of marginalized groups through measurement of SES with ecological measures alone may thus lead to an underestimation and/or under-adjustment of SES effects.

### Distribution of other patient characteristics by socioeconomic status

Individuals with financial hardship were more likely to be male, to live on their own, to have a disability and to present with a higher degree of illness severity as measured by the PSI. The independent association of all these variables with LOS confirms the importance of measuring these effects as potential confounders of socioeconomic status on hospital utilization.

### Study strengths and weaknesses

This study was limited by potential misclassification and unintended bias introduced by the retrospective nature of the data collection. It is possible for example, that there were those on social assistance or disability pensions, who for some reason, did not report this on admission or in the course of their clinical stay. If this was the case and these individuals had a disproportionate frequency of shorter lengths of stay or re-admissions, then the results may be confounded in a direction away from the null.

Another weakness is that we did not explore the reasons for individuals having missing postal code data at the time of chart review. This would be important to understand whether the missing data for this variable confounded the relation of neighbourhood income quintiles with our outcomes. However, our use of chart review data allowed us to employ both a more precise individual-level and an ecological SES measure, and compare the two. It also allowed us to use a richer set of clinical data to construct a disease-specific illness severity measure (such as PSI) and to examine other important potential confounding variables. The linkage of these chart review data with secondary provincial health data contributes to a more complete understanding of the relationship between SES and hospital utilization.

## Conclusion

Individuals with financial hardship have a longer hospital LOS and are more likely to experience re-admission to hospital, perhaps as a result of their social circumstances. However, there is no demonstrated income gradient associated with hospital LOS among patients admitted for pneumonia when income is measured as an ecological variable. The ecological income quintile variable, while useful in many circumstances, may be insufficiently sensitive to pick up SES effects in smaller clinical studies.

## Competing interests

The author(s) declare that they have no competing interests.

## Authors' contributions

MJM contributed to the research design, coordinated the implementation of the research and played a major role in the manuscript writing. RJR and ARL assisted with the study design and implementation. RJR, ARL and JMF all contributed to the data interpretation. MS supervised the data analysis and MBC performed the data analysis. All authors contributed to the manuscript writing and gave final approval of the version to be published.

## Pre-publication history

The pre-publication history for this paper can be accessed here:



## Figures and Tables

**Table 1:** Socioeconomic status and other characteristics of study sample (n = 434) §

**Characteristic**	**n (%)**
**Employable persons on income assistance **(C$7,081) ╪	84 (19)
**Persons receiving a provincial disability pension **(C$10,784) ╪	64 (15)
**Receiving income assistance or provincial disability pension**	148 (34)
**Neighbourhood-level income quintiles (average income range per single person equivalent)**╠	
1 (C$10,950 – C$29,001)	183 (45)
2 (C$29,037 – C$33,944)	79 (19)
3 (C$33,966 – C$38,834)	38 (9)
4 (C$38,881 – C$45,760)	45 (11)
5 (C$46,045 – C$122,256)	65 (16)
	
Missing	24
**Male**	189 (44)
**Mean Age, +/- SD**	45.1, +/-12.6
**Current smoker**	205 (47)
**Documented substance abuse issues**	111 (26)
**Pneumonia Severity Index**	
Mean, +/- SD	70.6, +/- 34.0
Median, min – max	64.0, 12 – 219
**Documented ADL problem**	19 (4)
**Number of co-morbidities**	
0	307 (71)
1	112 (26)
2	14 (3)
3	1 (0.2)
**Living alone**	107 (25)

§ Adult pneumonia admissions <65 years to Vancouver General Hospital, January 1, 1990 to March 31, 2001

╪ Annual benefits in British Columbia, 1996 [21]. These two categories of BC benefits are mutually exclusive; an individual cannot receive both at the same time. At ages 65 and over, individuals are no longer eligible for income assistance or provincial disability assistance.

╠ Range of 1996 census enumeration area average income per single person equivalent for Vancouver Census Metropolitan Area [18]

SD = standard deviation; min = minimum value; max = maximum value; ADL = activities of daily living

**Table 2:** Distribution of individual-level financial hardship by neighbourhood income quintiles among study sample (n = 434) §

**Neighbourhood-level income quintile**	**Individual-level financial hardship** ** n (%)**
1	78 (60)
2	16 (12)
3	15 (12)
4	12 (9)
5	8 (6)
	
Missing	19

§ Adult pneumonia admissions <65 years to Vancouver General Hospital, January 1, 1990 to March 31, 2001

**Table 3:** Distribution of length of stay predictors by socioeconomic status measure among study sample (n = 434) §

**Individual-level financial hardship**			
**Characteristic**	**Income assistance or provincial disability pension (n = 148)** ** n (%)**	**No income assistance or provincial disability pension (n = 286)** ** n (%)**	**p-value**

**Mean Age, +/- SD**	43.9, +/- 11.9	45.7, +/- 13.0	NS
**Male**	93 (63)	152 (53)	**0.05**
**Mean PSI, +/- SD**	75.5, +/- 37.4	68.1, +/- 31.8	**<0.05**
**Co-morbid Dx >1**	8 (5)	7 (2)	NS
**Documented ADL problem**	11 (7)	8 (3)	**<0.05**
**Living alone**	61 (41)	46 (16)	**<0.001**

**Neighbourhood-level income quintile**			

	**Quintile 1 (n = 183)**	**Quintiles 2–5 (n = 227)**	**p-value**

**Mean Age, +/- SD**	44.7, +/- 12.9	45.6, +/- 12.6	NS
**Male**	102 (56)	129 (57)	NS
**Mean PSI, +/- SD**	73.9, +/- 35.8	68.1, +/- 32.4	NS
**Co-morbid Dx > 1**	7 (4)	8 (4)	NS
**Documented ADL problem**	10 (5)	8 (4)	NS
**Living alone**	40 (22)	53 (23)	NS

§ Adult pneumonia admissions <65 years to Vancouver General Hospital, January 1, 1990 to March 31, 2001

SD = standard deviation; NS = not significant; PSI = pneumonia severity index;

Co-morbid Dx = co-morbidity diagnoses; ADL = activities of daily living

**Table 4:** Length of stay by socioeconomic status measure among study sample (n = 434) §

**SES measure**	**Mean LOS, +/- SD; Median LOS, min – max; n**
**Individual-level financial hardship**	
Yes	7.4, +/- 6.7; 6.0, 1 – 55; n = 148
No	5.4, +/- 4.3; 4.0, 1 – 36; n = 286
**p-value**	**<0.01**
**Neighbourhood-level income quintiles**	
1	6.2, +/- 5.2; 5.0, 1 – 33; n = 183
2	6.1, +/- 4.3; 5.0, 1 – 22; n = 79
3	5.2, +/- 2.9; 5.0, 1 – 15; n = 38
4	7.1, +/- 8.5; 5.0, 1 – 55; n = 45
5	5.7, +/- 5.4; 4.0, 1 – 36; n = 65
**p-value**	NS

§ Adult pneumonia admissions <65 years to Vancouver General Hospital, January 1, 1990 to March 31, 2001

**Table 5:** Adjusted percent differences (95% CI) in LOS by SES measure among study sample (n = 434) §

**SES measure**	**Adjusted % difference in LOS (95% CI)**	**p-value**	**R2 SES measure**	**R2 model**
**Individual-level financial hardship (n = 434) **╪				
Yes	15 (-0.4, +32)	**0.057**	0.028	0.245
No (reference)				
**Neighbourhood-level income quintiles (n = 410)**╠				
1	-11 (-27, +8)	NS	0.004	0.248
2	6 (-16, +33)	NS		
3	4 (-21, +37)	NS		
4	13 (-13, 46)	NS		
5 (reference)				

§ Adult pneumonia admissions <65 years to Vancouver General Hospital, January 1, 1990 to March 31, 2001

╪ Model adjusted for PSI, 15% (+11,+20); # co-morbidities, 24% (+10,+40); documented ADL problem, 85% (+34,+154); living alone, 20% (+3,+41); year of admission, -5% (-7,-3)

╠ Model adjusted for PSI, 15% (+11,+20); # co-morbidities, 24% (+9,+40); documented ADL problem, 96% (+42,+171); living alone, 24% (+5,+45); year of admission, -5% (-7,-3)

SES = socioeconomic status; LOS = length of stay; 95% CI = 95% confidence interval; NS = not significant; PSI = pneumonia severity index; ADL = activities of daily living

**Table 6:** Adjusted odds ratios (95% CI) for re-admission associated with SES measure among study sample (n=351) §

**SES measure**	**Adjusted OR (95% CI)**	**p-value**
**Individual-level financial hardship (n=351) **╪		
Yes	2.65 (1.38, 5.09)	**<0.01**
No (reference)		
**Neighbourhood-level income quintiles (n=335)**╠		
1	0.75 (0.29, 1.92)	NS
2	1.03 (0.39, 2.74)	NS
3	0.52 (0.13, 2.15)	NS
4	1.02 (0.29, 3.54)	NS
5 (reference)		

§ Adult pneumonia admissions <65 years to Vancouver General Hospital, January 1, 1990 to March 31, 2001 readmitted to hospital within 30 days of discharge

╪ Model adjusted for male sex, OR = 2.05 (1.01, 4.18)

╠ Model adjusted for male sex, OR = 2.15 (1.05, 4.40); # co-morbidities, OR = 1.72 (1.06, 2.79)
